# 1,3-Dimethyl 5-(4,4,5,5-tetra­methyl-1,3,2-dioxaborolan-2-yl)benzene-1,3-dicarboxyl­ate

**DOI:** 10.1107/S1600536811054559

**Published:** 2012-01-07

**Authors:** Long-tao Yi, Zhi-qiang Liu

**Affiliations:** a Department of Chemical and Biological Engineering, Zhejiang University, Hangzhou 310007, People’s Republic of China; b State Key Laboratory of Crystal Materials, Shandong University, Jinan 250100, People’s Republic of China

## Abstract

The title compound, C_16_H_21_BO_6_, has has approximate *C*_2_ symmetry, but no crystallographically imposed mol­ecular symmetry. In the crystal, mol­ecules are packed into parallel columns along the *a* axis. Short inter­molecular C—H⋯O contacts stabilize the crystal packing.

## Related literature

For the synthesis of organoboronic esters, see: Kikuchi *et al.* (2008 ▶). For the synthesis of the title compound, see: Coventry *et al.* (2005 ▶).
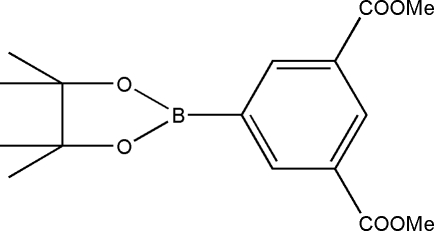


## Experimental

### 

#### Crystal data

C_16_H_21_BO_6_
*M* *_r_* = 320.14
Orthorhombic, 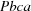

*a* = 7.2163 (2) Å
*b* = 20.9627 (4) Å
*c* = 22.4624 (4) Å
*V* = 3397.96 (13) Å^3^
*Z* = 8
Mo *K*α radiation
μ = 0.09 mm^−1^
*T* = 296 K
1.00 × 0.40 × 0.20 mm

#### Data collection

Bruker APEXII CCD diffractometer
Absorption correction: multi-scan (*SADABS*; Bruker, 2005 ▶) *T*_min_ = 0.766, *T*_max_ = 0.982
22413 measured reflections
3864 independent reflections
2177 reflections with *I* > 2σ(*I*)
*R*_int_ = 0.043

#### Refinement

*R*[*F* ^2^ > 2σ(*F* ^2^)] = 0.046
*wR*(*F* ^2^) = 0.142
*S* = 1.01
3864 reflections
215 parameters
H-atom parameters constrained
Δρ_max_ = 0.18 e Å^−3^
Δρ_min_ = −0.14 e Å^−3^

### 

Data collection: *APEX2* (Bruker, 2005 ▶); cell refinement: *SAINT* (Bruker, 2005 ▶); data reduction: *SAINT*; program(s) used to solve structure: *SHELXS97* (Sheldrick, 2008 ▶); program(s) used to refine structure: *SHELXL97* (Sheldrick, 2008 ▶); molecular graphics: *XP* in *SHELXTL* (Sheldrick, 2008 ▶); software used to prepare material for publication: *SHELXL97*.

## Supplementary Material

Crystal structure: contains datablock(s) I, global. DOI: 10.1107/S1600536811054559/bt5730sup1.cif

Structure factors: contains datablock(s) I. DOI: 10.1107/S1600536811054559/bt5730Isup4.hkl

Supplementary material file. DOI: 10.1107/S1600536811054559/bt5730Isup3.cml

Additional supplementary materials:  crystallographic information; 3D view; checkCIF report

## Figures and Tables

**Table 1:** Hydrogen-bond geometry (Å, °)

*D*—H⋯*A*	*D*—H	H⋯*A*	*D*⋯*A*	*D*—H⋯*A*
C16—H16*C*⋯O4^i^	0.96	2.53	3.381 (3)	148

Symmetry code: (i) 
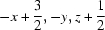
.

**Fig. 1.**
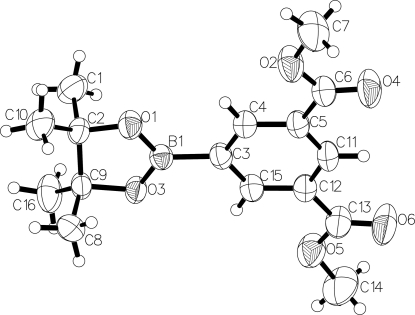
Molecular structure of the title compound. Displacement elliposoid is drawn at 50% probability level.

**Fig. 2.**
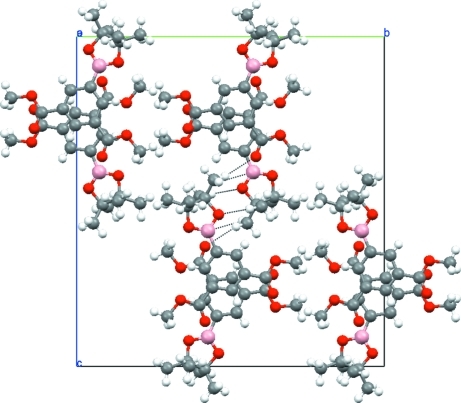
The packing pattern of the title molecules in crystal viewed down the a- axis. O···H and B···H intermolecular short contacts are also showed.

## supplementary crystallographic information

### Comment

Arylboronic acids and arylborates are the key reagents in Suzuki-Miyaura coupling reaction. Transition metal-catalyzed C—H borylation of arenes is a direct, economical and environmentally kind method to synthesize organoboronic esters and is widely studied (Kikuchi *et al.*, 2008). Recently, we have synthesized the title compound by direct Ir-catalyzed borylation and determined its X-ray structure.

The two *meta*-position methoxycarbonyl are almost co-planar with the benzene ring. The C—B bond is 1.556 (2) Å. Because the borolane ring adopts a somewhat twist conformation and C2, C9 atoms displace to opposite sides of the BO_2_ plane, the title molecule has no crystallographic symmetry.

The molecules pack into columns along the *a*- axis uniformly and exhibit paralled patterns. Besides, there are some short intermolecular O···H (for example, O3···H8a, O4···H16c distances are 2.636 Å, 2.530 Å, respectively). We believe that these short intermolecular contacts are helpful for stabilization of the molecular packing in crystals.

### Experimental

The title compound was synthesized *via* Ir-catalyzed borylation (Coventry *et al.*, 2005) of diethyl isophthalate. Catalyst precursor [Ir(COD)]Cl]_2_ (60 mg) and ligand dtbpy (4,4'-di-*tert*-bultyl-2,2'-dipyridyl) (120 mg) with a small amount of B_2_pin_2_ (pin=O_2_C_2_Me_4_) ((160 mg) were loaded in a Schlenk tube and dissolved in 2 ml THF under argon. The mixture was stirred vigorously till the bluish violet solution turned to amaranthine. Then diethyl isophthalate (1.94 g, 10.0 mmol) and B_2_pin_2_ (2.54 g, 10.0 mmol) in 15 ml THF was added into this tube. The mixture was heated in oil bath at 80°C for 24 h. After cooling to room temperature, the mixture then went through a short silica pad to remove the residual Ir catalyst and the final compound was purified by column chromatography using dichloromethane, giving 2.56 g (80% yield) white crystalline product. Crystals were grown by slow evaporation of a hexane solution.

### Crystal data

**Table:**

C_16_H_21_BO_6_	*F*(000) = 1360
*M**_r_* = 320.14	*D*_x_ = 1.252 Mg m^−^^3^
Orthorhombic, *P**b**c**a*	Mo *K*α radiation, λ = 0.71073 Å
Hall symbol: -P 2ac 2ab	Cell parameters from 3703 reflections
*a* = 7.2163 (2) Å	θ = 2.7–20.6°
*b* = 20.9627 (4) Å	µ = 0.09 mm^−^^1^
*c* = 22.4624 (4) Å	*T* = 296 K
*V* = 3397.96 (13) Å^3^	Pod, colourless
*Z* = 8	1.00 × 0.40 × 0.20 mm

### Data collection

**Table:**

Bruker APEXII CCD diffractometer	3864 independent reflections
Radiation source: fine-focus sealed tube	2177 reflections with *I* > 2σ(*I*)
graphite	*R*_int_ = 0.043
φ and ω scans	θ_max_ = 27.5°, θ_min_ = 1.8°
Absorption correction: multi-scan (*SADABS*; Bruker, 2005)	*h* = −9→9
*T*_min_ = 0.766, *T*_max_ = 0.982	*k* = −27→26
22413 measured reflections	*l* = −27→29

### Refinement

**Table:**

Refinement on *F*^2^	Secondary atom site location: difference Fourier map
Least-squares matrix: full	Hydrogen site location: inferred from neighbouring sites
*R*[*F*^2^ > 2σ(*F*^2^)] = 0.046	H-atom parameters constrained
*wR*(*F*^2^) = 0.142	*w* = 1/[σ^2^(*F*_o_^2^) + (0.0653*P*)^2^ + 0.3179*P*] where *P* = (*F*_o_^2^ + 2*F*_c_^2^)/3
*S* = 1.01	(Δ/σ)_max_ < 0.001
3864 reflections	Δρ_max_ = 0.18 e Å^−^^3^
215 parameters	Δρ_min_ = −0.14 e Å^−^^3^
0 restraints	Extinction correction: *SHELXL97* (Sheldrick, 2008), Fc^*^=kFc[1+0.001xFc^2^λ^3^/sin(2θ)]^-1/4^
Primary atom site location: structure-invariant direct methods	Extinction coefficient: 0.0031 (5)

### Special details

**Table:**

Experimental. SADABS (Bruker, 2005) was used for absorption correction. R(int) was 0.0776 before and 0.0475 after correction. The Ratio of minimum to maximum transmission is 0.9190. The λ/2 correction factor is Not present.
Geometry. All e.s.d.'s (except the e.s.d. in the dihedral angle between two l.s. planes) are estimated using the full covariance matrix. The cell e.s.d.'s are taken into account individually in the estimation of e.s.d.'s in distances, angles and torsion angles; correlations between e.s.d.'s in cell parameters are only used when they are defined by crystal symmetry. An approximate (isotropic) treatment of cell e.s.d.'s is used for estimating e.s.d.'s involving l.s. planes.
Refinement. Refinement of *F*^2^ against ALL reflections. The weighted *R*-factor *wR* and goodness of fit *S* are based on *F*^2^, conventional *R*-factors *R* are based on *F*, with *F* set to zero for negative *F*^2^. The threshold expression of *F*^2^ > σ(*F*^2^) is used only for calculating *R*-factors(gt) *etc*. and is not relevant to the choice of reflections for refinement. *R*-factors based on *F*^2^ are statistically about twice as large as those based on *F*, and *R*- factors based on ALL data will be even larger.

### Fractional atomic coordinates and isotropic or equivalent isotropic displacement parameters (Å^2^)

**Table:**

	*x*	*y*	*z*	*U*_iso_*/*U*_eq_	
O1	0.67152 (17)	0.13558 (5)	0.42187 (5)	0.0525 (4)	
O2	0.6570 (2)	0.16197 (6)	0.20099 (6)	0.0739 (5)	
O3	0.74902 (17)	0.03889 (5)	0.46030 (5)	0.0484 (3)	
O4	0.6855 (2)	0.08265 (7)	0.13639 (6)	0.0817 (5)	
O5	0.8187 (2)	−0.14641 (6)	0.32004 (7)	0.0785 (5)	
O6	0.8121 (3)	−0.13786 (7)	0.22123 (7)	0.0903 (6)	
C1	0.4427 (3)	0.13373 (9)	0.49842 (10)	0.0679 (6)	
H1A	0.3750	0.1642	0.4751	0.102*	
H1B	0.4192	0.1411	0.5399	0.102*	
H1C	0.4037	0.0914	0.4880	0.102*	
C2	0.6479 (2)	0.14086 (7)	0.48620 (8)	0.0439 (4)	
C3	0.7180 (2)	0.04498 (8)	0.34685 (7)	0.0452 (4)	
C4	0.6938 (2)	0.08372 (9)	0.29731 (8)	0.0474 (4)	
H4	0.6698	0.1269	0.3028	0.057*	
C5	0.7043 (2)	0.05958 (8)	0.23990 (8)	0.0462 (4)	
C6	0.6823 (3)	0.10082 (9)	0.18671 (9)	0.0546 (5)	
C7	0.6358 (4)	0.20504 (11)	0.15139 (9)	0.0952 (9)	
H7A	0.7436	0.2027	0.1264	0.143*	
H7B	0.6216	0.2478	0.1659	0.143*	
H7C	0.5282	0.1933	0.1288	0.143*	
C8	0.9702 (3)	0.10025 (10)	0.51525 (9)	0.0702 (6)	
H8A	1.0391	0.0619	0.5225	0.105*	
H8B	0.9880	0.1293	0.5478	0.105*	
H8C	1.0128	0.1197	0.4790	0.105*	
C9	0.7647 (3)	0.08423 (8)	0.50955 (7)	0.0461 (4)	
C10	0.7157 (3)	0.20608 (8)	0.50516 (9)	0.0651 (6)	
H10A	0.8413	0.2118	0.4922	0.098*	
H10B	0.7102	0.2094	0.5477	0.098*	
H10C	0.6386	0.2383	0.4876	0.098*	
C11	0.7376 (3)	−0.00497 (9)	0.23160 (8)	0.0515 (5)	
H11	0.7437	−0.0216	0.1933	0.062*	
C12	0.7619 (2)	−0.04497 (8)	0.28033 (8)	0.0481 (5)	
C13	0.7995 (3)	−0.11372 (10)	0.26942 (10)	0.0621 (6)	
C14	0.8615 (5)	−0.21352 (10)	0.31398 (12)	0.1040 (9)	
H14A	0.7605	−0.2348	0.2945	0.156*	
H14B	0.8803	−0.2318	0.3527	0.156*	
H14C	0.9721	−0.2184	0.2907	0.156*	
C15	0.7531 (2)	−0.01959 (8)	0.33725 (8)	0.0488 (5)	
H15	0.7709	−0.0463	0.3698	0.059*	
C16	0.6931 (4)	0.05342 (10)	0.56561 (9)	0.0764 (7)	
H16A	0.5692	0.0383	0.5591	0.115*	
H16B	0.6928	0.0841	0.5973	0.115*	
H16C	0.7717	0.0182	0.5761	0.115*	
B1	0.7131 (3)	0.07327 (9)	0.41089 (9)	0.0421 (5)	

### Atomic displacement parameters (Å^2^)

**Table:**

	*U*^11^	*U*^22^	*U*^33^	*U*^12^	*U*^13^	*U*^23^
O1	0.0751 (9)	0.0423 (7)	0.0400 (7)	0.0040 (6)	−0.0016 (6)	−0.0003 (5)
O2	0.1243 (13)	0.0555 (9)	0.0418 (8)	−0.0010 (8)	−0.0037 (8)	0.0037 (6)
O3	0.0700 (9)	0.0377 (6)	0.0375 (7)	0.0065 (5)	−0.0026 (6)	−0.0054 (5)
O4	0.1359 (14)	0.0728 (10)	0.0364 (8)	0.0053 (9)	0.0016 (8)	−0.0029 (7)
O5	0.1204 (13)	0.0542 (9)	0.0610 (10)	0.0147 (8)	0.0004 (9)	−0.0028 (7)
O6	0.1523 (17)	0.0612 (9)	0.0573 (10)	0.0077 (9)	0.0085 (10)	−0.0174 (7)
C1	0.0513 (12)	0.0673 (13)	0.0853 (16)	0.0041 (10)	0.0048 (11)	−0.0188 (11)
C2	0.0520 (11)	0.0382 (9)	0.0417 (10)	0.0004 (7)	0.0012 (8)	−0.0070 (7)
C3	0.0483 (10)	0.0526 (10)	0.0349 (10)	−0.0010 (8)	0.0002 (8)	−0.0016 (8)
C4	0.0514 (11)	0.0490 (10)	0.0419 (10)	−0.0024 (8)	0.0019 (8)	−0.0032 (8)
C5	0.0492 (11)	0.0538 (11)	0.0357 (10)	−0.0045 (8)	0.0019 (8)	−0.0023 (8)
C6	0.0643 (13)	0.0580 (12)	0.0415 (12)	−0.0046 (9)	0.0020 (9)	−0.0024 (9)
C7	0.165 (3)	0.0633 (14)	0.0578 (15)	0.0008 (15)	−0.0084 (15)	0.0172 (12)
C8	0.0604 (14)	0.0768 (14)	0.0736 (15)	0.0113 (10)	−0.0213 (11)	−0.0182 (11)
C9	0.0623 (12)	0.0421 (9)	0.0337 (10)	0.0029 (8)	−0.0058 (8)	−0.0074 (7)
C10	0.0740 (14)	0.0416 (10)	0.0797 (16)	−0.0033 (9)	−0.0017 (11)	−0.0137 (10)
C11	0.0601 (12)	0.0596 (11)	0.0349 (10)	−0.0051 (9)	0.0023 (8)	−0.0088 (9)
C12	0.0517 (11)	0.0502 (10)	0.0424 (11)	−0.0024 (8)	0.0007 (8)	−0.0064 (8)
C13	0.0777 (15)	0.0574 (12)	0.0514 (13)	−0.0001 (10)	0.0035 (11)	−0.0059 (10)
C14	0.163 (3)	0.0530 (14)	0.096 (2)	0.0214 (15)	0.0036 (18)	0.0028 (13)
C15	0.0546 (11)	0.0509 (11)	0.0410 (10)	−0.0012 (8)	0.0001 (8)	−0.0019 (8)
C16	0.128 (2)	0.0618 (13)	0.0393 (12)	0.0006 (12)	0.0063 (12)	0.0024 (10)
B1	0.0470 (11)	0.0404 (10)	0.0390 (12)	−0.0019 (8)	−0.0001 (9)	0.0004 (9)

### Geometric parameters (Å, °)

**Table:**

O1—B1	1.363 (2)	C7—H7A	0.9600
O1—C2	1.459 (2)	C7—H7B	0.9600
O2—C6	1.334 (2)	C7—H7C	0.9600
O2—C7	1.442 (2)	C8—C9	1.526 (3)
O3—B1	1.349 (2)	C8—H8A	0.9600
O3—C9	1.4629 (19)	C8—H8B	0.9600
O4—C6	1.193 (2)	C8—H8C	0.9600
O5—C13	1.335 (2)	C9—C16	1.506 (3)
O5—C14	1.447 (2)	C10—H10A	0.9600
O6—C13	1.198 (2)	C10—H10B	0.9600
C1—C2	1.514 (3)	C10—H10C	0.9600
C1—H1A	0.9600	C11—C12	1.390 (2)
C1—H1B	0.9600	C11—H11	0.9300
C1—H1C	0.9600	C12—C15	1.386 (2)
C2—C10	1.513 (2)	C12—C13	1.487 (3)
C2—C9	1.547 (2)	C14—H14A	0.9600
C3—C4	1.389 (2)	C14—H14B	0.9600
C3—C15	1.394 (2)	C14—H14C	0.9600
C3—B1	1.556 (2)	C15—H15	0.9300
C4—C5	1.387 (2)	C16—H16A	0.9600
C4—H4	0.9300	C16—H16B	0.9600
C5—C11	1.387 (3)	C16—H16C	0.9600
C5—C6	1.483 (3)		
			
B1—O1—C2	106.12 (12)	O3—C9—C16	109.10 (14)
C6—O2—C7	115.49 (16)	O3—C9—C8	106.34 (14)
B1—O3—C9	106.86 (13)	C16—C9—C8	110.94 (17)
C13—O5—C14	116.17 (17)	O3—C9—C2	101.54 (12)
C2—C1—H1A	109.5	C16—C9—C2	115.20 (16)
C2—C1—H1B	109.5	C8—C9—C2	112.88 (15)
H1A—C1—H1B	109.5	C2—C10—H10A	109.5
C2—C1—H1C	109.5	C2—C10—H10B	109.5
H1A—C1—H1C	109.5	H10A—C10—H10B	109.5
H1B—C1—H1C	109.5	C2—C10—H10C	109.5
O1—C2—C10	108.03 (14)	H10A—C10—H10C	109.5
O1—C2—C1	106.63 (14)	H10B—C10—H10C	109.5
C10—C2—C1	110.76 (15)	C5—C11—C12	120.31 (16)
O1—C2—C9	102.36 (12)	C5—C11—H11	119.8
C10—C2—C9	114.95 (15)	C12—C11—H11	119.8
C1—C2—C9	113.30 (15)	C15—C12—C11	119.27 (17)
C4—C3—C15	117.82 (16)	C15—C12—C13	122.17 (17)
C4—C3—B1	121.00 (16)	C11—C12—C13	118.56 (17)
C15—C3—B1	121.15 (16)	O6—C13—O5	123.02 (19)
C5—C4—C3	121.65 (17)	O6—C13—C12	124.89 (19)
C5—C4—H4	119.2	O5—C13—C12	112.09 (17)
C3—C4—H4	119.2	O5—C14—H14A	109.5
C11—C5—C4	119.35 (16)	O5—C14—H14B	109.5
C11—C5—C6	118.62 (16)	H14A—C14—H14B	109.5
C4—C5—C6	122.02 (16)	O5—C14—H14C	109.5
O4—C6—O2	122.50 (18)	H14A—C14—H14C	109.5
O4—C6—C5	125.10 (18)	H14B—C14—H14C	109.5
O2—C6—C5	112.40 (16)	C12—C15—C3	121.59 (17)
O2—C7—H7A	109.5	C12—C15—H15	119.2
O2—C7—H7B	109.5	C3—C15—H15	119.2
H7A—C7—H7B	109.5	C9—C16—H16A	109.5
O2—C7—H7C	109.5	C9—C16—H16B	109.5
H7A—C7—H7C	109.5	H16A—C16—H16B	109.5
H7B—C7—H7C	109.5	C9—C16—H16C	109.5
C9—C8—H8A	109.5	H16A—C16—H16C	109.5
C9—C8—H8B	109.5	H16B—C16—H16C	109.5
H8A—C8—H8B	109.5	O3—B1—O1	113.93 (15)
C9—C8—H8C	109.5	O3—B1—C3	123.55 (16)
H8A—C8—H8C	109.5	O1—B1—C3	122.52 (16)
H8B—C8—H8C	109.5		

### Hydrogen-bond geometry (Å, °)

**Table:**

*D*—H···*A*	*D*—H	H···*A*	*D*···*A*	*D*—H···*A*
C16—H16C···O4^i^	0.96	2.53	3.381 (3)	148

Symmetry codes: (i) −*x*+3/2, −*y*, *z*+1/2.
